# Association of serotonin transporter gene polymorphisms with obsessive-compulsive disorder (OCD) in a south Indian population

**Published:** 2010-12

**Authors:** Prashant Tibrewal, Kumar H.B. Kiran, G.N. Shubha, D. Subhashree, Meera Purushottam, K. Thennarasu, Y.C.J Reddy, Sanjeev Jain

**Affiliations:** *+ Department of Psychiatry, National Institute of Mental Health & Neurosciences, Bangalore, India; * Department of Biostatistics, National Institute of Mental Health & Neurosciences, Bangalore, India; ** Department of Biology, The University of Western Ontario, London, Canada

**Keywords:** 5-HTTVNTR and 5-HTTLPR polymorphisms, obsessive-compulsive disorder (OCD), serotonin transporter (5-HTT, SLC6A4)

## Abstract

**Background and Objectives:**

Serotonin transporter polymorphisms, 5-HTTVNTR and 5-HTTLPR, have been found to be associated with obsessive-compulsive disorder (OCD) and particularly with neurotic characteristics. In the present study we looked for an association between OCD and these polymorphisms in OCD patients and controls of south Indian origin.

**Methods:**

5-HTTVNTR and 5-HTTLPR/rs25531 were genotyped in 93 OCD patients and 92 healthy controls. The allelic distribution and genotype frequency in cases and controls were compared using chi square test. In order to test for the effects of genotype on heterogeneity of the illness, linear regression analysis was undertaken for co-morbid depression status and YBOCS score (severity index).

**Results:**

There was no significant association with the 5-HTTVNTR or the 5-HTTLPR polymorphism. No significant association of OCD with the 5-HTTLPR genotype was found even on inclusion of the rs25531 locus, which is part of the transcription factor binding site as reported in earlier studies. However, severity of the illness showed a modest association with the dominant model.

**Interpretation & conclusions:**

Our data show that genetic variation in the *SLC6A4* gene regulatory region may not have a significant effect on OCD in the present population. Further replication in a large and independent cohort with an equal number of female subjects would help to ascertain if the absence of association in this cohort is due to the nullifying effect of the larger proportion of male subjects in our sample population. The marginal effect of the 5-HTTLPR (A/G) genotype obtained on linear regression with disease severity is suggestive of a potential role for this locus in the disease process.

Obsessive compulsive disorder (OCD) is the fourth most prevalent psychiatric disorder with a lifetime prevalence of 2-3 per cent^1^. It is often a chronic illness characterized by obsessional thoughts and/or compulsive acts^2^. Although OCD is often considered a unitary disorder, there is evidence that it is a heterogeneous disorder^3^. Juvenile OCD and tic related OCD are considered to be valid subtypes^4^–^6^.

Good therapeutic efficacy of serotonin reuptake inhibitors (SRIs) in OCD^7^,^8^ has prompted search for association between OCD and variations in the *SLC6A4* gene. A functional polymorphism in the 5' regulatory promoter region 5-HTTLPR (5-HTT gene-linked polymorphic region), involving two common alleles that correspond to a 43-base pair insertion (L allele) or deletion (S allele), has been reported^9^. The S allele of 5-HTTLPR polymorphism reduces transcription efficiency for the *SLC6A4* gene, resulting in decreased gene expression, and thus, decreased serotonin uptake in lymphoblast cell lines^10^. Recent research shows varied results with respect to association between 5-HTTLPR alleles and OCD. In a meta-analysis by Lin^11^, only two studies^12^,^13^ showed a significant association of LL genotype with OCD. The overall result of the meta-analysis demonstrated an association of OCD with SS genotype and a reverse association with L/S genotype. Denys *et al*^14^, found an association of S allele of 5-HTTLPR with female OCD patients. However, Dickel *et al*^15^, supported a nominally significant over-transmission of the L allele in female patients. A recent meta-analysis suggests the possibility that the L allele may be associated with OCD in specific subgroups such as childhood-onset OCD, and in Caucasians^16^.

A possible association between the Stin2.12 allele of the other polymorphism 5-HTTVNTR in the second intron of *SLC6A4* and OCD has been suggested^17^,^18^. Role of combined effect of 5-HTTLPR and VNTR polymorphism on the expression of the serotonin transporter has also been reported^19^.

Hu *et al*^13^ demonstrated that possible lack of consistent association with 5-HTTLPR was the overlooked contribution of a single nucleotide polymorphism (SNP), rs25531, within the repetitive region that comprises the 5-HTTLPR. The modulation of 5-HTTLPR by rs25531 results in three common alleles: L_A_ (highest-expressing), _-_L_G_ and S (both low expressing). The L_G_ allele creates a functional AP2 transcription-factor binding site which is one of the nuclear factors that function as transcriptional activators or repressors^20^ and reduces transcription of the transporter protein. They found an association in two independent samples, both case-control and family-based, of the higher-expressing L_A_ allele and L_A_L_A_ genotype with OCD.

Replication in a large case-control design was unable to corroborate this, although an increased frequency of the L_A_ allele and L_A_L_A_ genotype in OCD probands was observed^21^. Wendland *et al*^22^ reported a highly significant haplotype-based omnibus association of all the known non-coding functional SLC6A4 variants with OCD in a large case-control sample. The haplotype significantly overrepresented in probands contained the higher-expressing L allele at each locus, supporting the notion of increased serotonin transporter functioning being pathogenetically involved in OCD.

Existing evidences of involvement of *SLC6A4* in OCD, prompted us to study the polymorphisms at this region in a south Indian population of OCD subjects and controls. To reduce the phenotypic heterogeneity, we included only adults with OCD who did not have co-morbid tic disorder.

## Material & Methods

The institutional Ethics Committee approved the study protocol. Participants gave written informed consent for participation in the study.

Subjects: Consecutive subjects fulfilling DSM-IV criteria for OCD^23^ were recruited from the OCD Clinic and the clinical services of the National Institute of Mental Health and Neurosciences (NIMHANS), Bangalore during April 2006 to January 2008. Those with a history of substance abuse and co-morbid tic disorder were excluded. All patients and controls were adults, unrelated and of south Indian origin. The Mini-International Neuropsychiatric Interview (MINI) version 4.4, a brief structured interview^24^, was used for the DSM-IV diagnosis of 93 OCD patients, and for the exclusion of any Axis-I diagnosis in 92 healthy controls. All patients were further assessed using the Yale-Brown Obsessive Compulsive Scale (YBOCS) symptom checklist and Y-BOCS severity rating scale for symptom profile and severity of OCD^25^,^26^. Clinical Global Impression CGI-S and CGI-I^27^ were applied to assess the global severity and improvement. The patients and controls were matched for age and gender.

The 5-HTTVNTR, and 5-HTTLPR (A/G) polymorphism was genotyped in OCD patients (n=93) and healthy controls (n= 92). The demographic and clinical details of the case control sample are shown in Table I.

**Table I:** Demographic information of the case-control sample set

	No. (%)
*Cases* (N= 92):	
Mean age (yr)	28.8 ± 9.04
Number of males	62
Age of onset (yr)	21.14 ± 7.98
Onset before 18 yr of age	42 ± 4
Duration of illness (month)	98.59 ± 9.5
Duration of untreated illness (month)	53.13 ± 7.0
Family history of any psychiatric illness	42 (44.2)
(i) OCD	13 (13.7)
(ii) Substance abuse	3 (3.2)
(iii) Psychosis	11 (11.6)
(iv) Affective illness	12 (12.6)
(v) Anxiety disorder	3 (3.2)
(vi) Suicide	10 (10.5)
(vii) Others	5 (5.3)
*Controls* (N=92):	
Age (yr)	29.47 ± 9.5
Number of males	62

*DNA methods*: Venous blood (10 ml) was drawn from patients and controls under aseptic precautions. DNA was isolated from peripheral leucocytes using salting out method^28^. Oligonucleotide primers flanking the 5-HTTVNTR were used to generate fragments corresponding to the 12 and 10 alleles^29^. The 5-HTTLPR/ (A/G) polymorphisms were genotyped as described in Wendland *et al*^30^. Five unlinked Alu polymorphisms were genotyped in OCD patients (n=93), and control (n=50) subjects to rule out the impact of stratification in the case-control association study. There was no differential distribution of alleles between the cases and controls at any of the Alu loci ruling out population stratification in the cohort (data not shown).

*Statistical analysis*: The allelic distribution and genotype frequency in cases and controls were compared using chi square test after checking the Hardy-Weinberg Equilibrium. Logistic regression was applied to test for effects of confounding factors. In order to test for the effects of genotype on heterogeneity of the illness, linear regression was performed (using SPSS v13.0) on the 5-HTTLPR and rs25531 genotype data with severity index and co-morbid depression as dependent variable and the genotypes as independent variables. The effect of genotype 5-HTTLPR(A/G) on YBOCS score was assessed by testing the regression coefficient (β) under three different genetic models separately with two sided test. Three models dominant, co-dominant and recessive were assumed with the genotypes at both loci. Haplotype based association analysis was carried out using the 2LD calculator^31^.

## Results

There was no deviation from Hardy-Weinberg Equilibrium at either of the polymorphisms studied. There was no association of the 5-HTTVNTR polymorphisms of the *SLC6A4* gene both with the genotypic and allelic frequencies between OCD patients and healthy control samples (Table II).

**Table II:** Genotypic and allelic distribution at serotonin transporter loci

	Genotype frequency			Allele frequency		
	Controls (n=92)	OCD cases (n=93)		Controls (n=184)	OCD cases (n=186)
*Genotypic and allelic distribution at 5-HTTVNTR:*					
10 10	12 (0.103)	11 (0.118)	10	55 (0.298)	52 (0.279)
10 12	31 (0.336)	30 (0.322)	12	129 (0.701)	134 (0.720)
12 12	49 (0.532)	52 (0.559)			
	Chi square=0.14 df=2 *P*=0.93			Chi square=.09 df=1 *P=0.76* Genotypic		
*Genotypic and allelic distribution of 5-HTTLPR (L/S) polymorphism and rs25531(A/G):*					
L_A_ L_A_	4 (0.04)	8 (0.08)	L_A_	47 (0.25)	53 (0.28)
L_A_ L_G_	6 (0.06)	7 (0.07)	L_G_	25 (0.13)	16 (0.08)
L_G_ L_G_	1 (0.01)	1 (0.01)	S	112 (0.60)	117 (0.63)
S L_A_	33 (0.35)	30 (0.32)			
S L_G_	17 (0.18)	7 (0.075)			
SS	31 (0.33)	40 (0.43)			
	Chi square=6.855 df=5 *P*=0.23			Chi square=2.43 df=2 *P*=0.296		
*Expression based grouping of 5-HTTLPR (L/S) polymorphism and rs25531(A/G) genotypes:*					
SS, SL_G_, L_G_ L_G_ (Low)	49 (0.53)	48 (0.51)	S or L_G_ (Low)	137 (0.74)	133 (0.71)
L_A_ L_G_, SL_A_ (Medium)	39 (0.42)	37 (0.39)	L_A_ (High)	47 (0.25)	53 (0.28)
L_A_ L_A_ (High)	4 (0.04)	8 (0.08)			
	Chi square=1.39 df=2 *P*=0.49			Chi square=0.27 df=1 *P*=0.6		

Since prior evidence from gene expression studies indicated that the “low expressing” SL_G_ and SS genotypes are not distinguishable biochemically, the distribution of the L_A_L_A_, S/L_A_, L_A_L_G_ and the combined SL_G_ and SS genotypes was compared between case and controls, but there was no significant difference (Table II). The allele frequencies were found to differ between the patients and controls with a modest higher frequency of the L_A_ allele in the cases compared with the controls (28% vs. 25%); further, the frequency of L_G_ allele differed between the cases and controls (8% vs 13%). However, none of these differences were statistically significant. Logistic regression was applied to test for effects of age and sex. No significant contribution was seen on assuming any of the three inheritance models (data not shown). No significant difference was found in frequencies of haplotypes in cases and controls (Table III).

**Table III:** Estimated haplotype frequencies of the 5-HTTVNTR and 5-HTTLPR polymorphisms

	5HTTLPR				
	5HTTVNTR	(L_A_)	(L_G_)	(S_A_)	(S_G_)
Controls	10	0.1430	0.0311	0.5380	0.0107
	12	0.1450	0.0504	0.0761	0.0056
Cases	10	0.1246	0.0784	0.4959	0.0000
	12	0.1281	0.0614	0.1008	0.0108
Combined	10	0.1325	0.0568	0.5180	0.0035
	12	0.1378	0.0540	0.0874	0.0100

**Table IV:** Linear regression analysis with severity index as dependent and 5-HTTLPR (A/G) genotype as independent variable

Genetic model	Beta	Std error	t	*P* value
Dominant	0.23	1.85	2.14	0.036*
Co-dominant	0.17	0.84	1.53	0.13
Recessive	0.09	1.14	0.78	0.44

* Significant

On clinical evaluation, 58 per cent of the cases had a co-morbid psychiatric illness, of which 41 per cent had major depression. There was a family history of psychiatric illness in 44 per cent of cases and family history of OCD was found in 13 per cent. There was no significant difference in the allele or genotype frequencies in any of the above subgroups.

On linear regression analysis, there was no significant association of the 5-HTTLPR locus with both phenotypes (severity index and co-morbid depression). However, a statistically significant association (*P*=0.036) of the dominant model of 5-HTTLPR (A/G) (non-risk allele: L_A_; risk alleles: S_A_, S_G_, L_G_) was seen with the severity index or YBOCS score (Table IV). Similar analysis for association of the SNP with co-morbid depression did not yield any significant result (data not shown).

## Discussion

In the present study involvement of the 5-HTTVNTR and 5-HTTLPR (A/G) gene polymorphism with OCD was examined. There was no association with the 5-HTTVNTR polymorphism. At the 5-HTTLPR (A/G) locus no significant association was observed. Also no significant association was observed on comparing the combined frequencies of lower expressing genotypes (SS, SL, L_G_ L_G_) and intermediate expressing genotypes (SL_A_, L_A_L_G_) with higher expressing (L_A_L_A_) genotypes. However, there was a modest association of the dominant model of 5-HTTLPR (A/G) with the severity index of OCD. This may indicate that the promoter SNP rs25531 which has a role in the expression efficiency of the gene (*SLC6A4*), may partly mediate the pathophysiology of OCD at least in terms of severity of symptoms.

The S allele frequency in our control population (0.60) was more than that reported in White European origin populations (0.35-0.40)^13^. However, it varies across other populations, varying from 0.454 in a Brazilian sample to 0.64-0.66 in American Indians^32^. It is noteworthy that in the populations which have shown an association of L allele with OCD, the L allele frequency was higher (0.592 and 0.60 to 0.65 respectively)^12^,^13^ than in our sample (L= 0.39).

Association analyses of common functional variants of the *SLC6A4* gene, a long-standing OCD candidate, have so far been inconsistent. Our findings lend support for the putative association of serotonin transporter variations to severity of OCD. The variation in allele frequencies across populations with possible different contributions to disease phenotypes across ethnicities underlines the need for comparative studies, at both phenotypic and genotypic levels, to further explore the biology of this syndrome. Genotyping additional SNPs/novel SNPs around the complex 5-HTTLPR and 5-HTTVNTR region and inclusion of these in LD (Linkage disequilibrium) based association will facilitate identification of risk alleles/haplotypes for OCD in the population.

One possible explanation for the equivocal results across populations could be the presence of sex-related differences and co-morbid diagnosis. Effect of gender has been reported in OCD previously^33^,^34^. In our sample cohort, the representation of males in cases and controls was high. However, the small sample size could be a confounding reason responsible for lack of significance on logistic regression. The small size and the consequent lack of statistical power is the major limitation of our study. Thus, further examination of these loci on a larger sample set with adoption of a family based association design may also help rule out other confounding factors like ethnicity.

The results from this study and earlier association studies indicate that OCD resembles other complex psychiatric disorders in being aetiologically heterogeneous. It is possible that the modest significance derived on linear regression with severity is a reflection of such a phenomenon. Genetic contributions may involve highly penetrant alleles and/or more common rare alleles, as well as a polygenetic inheritance involving multiple polymorphisms in serotonergic candidate genes. The understanding of these contributions requires further studies under the triallelic model and replication in a larger sample size.
